# Genome-wide identification and characterization of Glyceraldehyde-3-phosphate dehydrogenase genes family in wheat (*Triticum aestivum*)

**DOI:** 10.1186/s12864-016-2527-3

**Published:** 2016-03-16

**Authors:** Lingfeng Zeng, Rong Deng, Ziping Guo, Shushen Yang, Xiping Deng

**Affiliations:** College of Life Sciences, Northwest A&F University, 712100 Yangling, Shaanxi PR China; Institute of Soil and Water Conservation, Chinese Academy of Sciences, 712100 Yangling, Shaanxi PR China

**Keywords:** Wheat, *Triticum aestivum*, Glyceraldehyde-3-phosphate dehydrogenase, Expression profiles, Abiotic stress responses

## Abstract

**Background:**

Glyceraldehyde-3-phosphate dehydrogenase (GAPDH) is a central enzyme in glycolysi, we performed genome-wide identification of *GAPDH* genes in wheat and analyzed their structural characteristics and expression patterns under abiotic stress in wheat.

**Results:**

A total of 22 *GAPDH* genes were identified in wheat cv. Chinese spring; the phylogenetic and structure analysis showed that these *GAPDH* genes could be divided into four distinct subfamilies. The expression profiles of *GAPDH* genes showed tissue specificity all over plant development stages. The qRT-PCR results revealed that wheat *GAPDH*s were involved in several abiotic stress response.

**Conclusions:**

Wheat carried 22 *GAPDH* genes, representing four types of plant *GAPDH*s (*gapA*/*B*, *gapC*, *gapCp* and *gapN*). Whole genome duplication and segmental duplication might account for the expansion of wheat *GAPDH*s. Expression analysis implied that GAPDHs play roles in plants abiotic stress tolerance.

**Electronic supplementary material:**

The online version of this article (doi:10.1186/s12864-016-2527-3) contains supplementary material, which is available to authorized users.

## Background

Glyceraldehyde-3-phosphate dehydrogenase (GAPDH), catalyzing the conversion of glyceraldehyde-3-phosphate (G3P) to 1,3-biphosphoglycerate in the presence of NAD^+^ and inorganic phosphate, is a key enzyme in the glycolytic pathway [1]. GAPDH was once considered as a simple “housekeeping gene” and has been used as a reference in gene expression and protein studies [2]. However, it’s recently demonstrated that GAPDH plays crucial roles in many cellular processes in addition to glycolysis [3–5].

As a ubiquitous enzyme, GAPDH exists in nearly all organisms. In plant cells, GAPDHs, according to their different subcellular locations, could be classified into three groups as follows: GAPA/B, encoded by *gapA* and *gapB*, chloroplastic phosphorylating NADP-specific GAPDHs participating in photosynthetic CO_2_ fixation; GAPC, from gene *gapC*, a phosphorylating NAD-dependent GAPDH catalyzing the conversion of glyceraldehyde-3-P (Ga3P) to 1,3-bisphosphoglycerate in cytoplasm; GAPCp, encoded by *gapCp*, involved in glycolytic energy production in non-green plastids [6–14]. Depending on species, each type of *gap* gene may be further duplicated, for example, Arabidopsis contains two *gapA*, two *gapC* and two *gapCp* genes, but only a single *gapB* [15, 16]. In addition, plant contains a cytosolic non-phosphorylating NADP-dependent GAPDH (GAPN), which belongs to the aldehyde dehydrogenase superfamily and has no close functional and structural relationships with phosphorylating GAPDHs, catalyzing the oxidation of Ga3P to 3-phosphoglycerate (3PGA) [17–21].

GAPDH is essentially a tetramer of identical or similar subunits. The glycolytic GAPDHs (GAPC, GAPCp) and A4-GAPDH of oxygenic phototrophs compose of identical subunits, while the main photosynthetic isoform of land plants (A2B2-GAPDH) consist of similar subunits. The main features of GAPDH protein monomers are highly conserved in all living organisms, consisting of an N-terminal NAD (P)-binding domain called Gp_dh_N domain (PF00044) and a C-terminal catalytic domain named Gp_dh_C domain (PF02800). Some GAPDH genes (AtGAPB) contain an extra incomplete CP12 domain (PF02672). While GAPNs contain an Adledh domain.

Besides its pivotal role in glycolysis, GAPDH is a moonlighting protein [13]. In Mammals, GAPDH participatosis [1, 4, 5, 22] and cell toxicity [23]. Nonglycolytic functiones in signal transduction cascades, DNA repair, transcriptional regulation, apopts of plant GAPDH have been demonstrated as well, especially in abiotic stress responses. For instance, In Aspen (*Populus tremula*), GAPDH showed some increase in response to water deficit [24]. Overexpression of a cytosolic *OsGAPC3* enhanced salt tolerance in rice [25]. Furthermore, GAPDH generated immunocomplex with NtOSAK (osmotic stress-activated protein kinase) in salt-treated tobacco cells [26]. Moreover, GAPDH interacted with Phospholipase Dδ (PLDδ) to transduce hydrogen peroxide signals and the GAPC–PLDδ interaction mediated response to ABA and water deficits in Arabidopsis [27]. Besides abiotic stress responses, plant GAPDHs have been implicated in embryo development, pollen development, root development [16, 28–31].

Although vast studies have been conducted on biochemical properties and physiological functions of GAPDHs, few systematic research on the evolution and functional divergence of *GAPDH* gene family have been conducted, especially in wheat (*Triticum aestivum*). Recent plant genome sequencing projects have strongly promoted the identification and characterization of plant genes. Wheat genome sequencing is now nearing completion (http://www.wheatgenome.org/). Hence, a comprehensive analysis of the wheat *GAPDH* family was conducted here. We systematically identified and characterized the *GPADH* genes of wheat (*TaGAPDH*) and compared them with *GAPDH* in *Arabidopsis thaliana*, *Hordeum vulgare*, *Aegilops tauschii* and *Triticum urartu*. In addition, the expression profiles of *GAPDH* genes in different tissues and at different stages as well in response to several stress were analyzed. Finally, the inducible expression of wheat *GAPDH* genes were detected by qRT-PCR experiments, and some *GAPDH*s were found notably responding to abiotic stress.

## Results

### Identification, phylogenetic analysis and classification of wheat *GAPDH* genes

By Hidden Markov model (HMM) searches on the available *Triticum aestivum* sequenced genome, 40 sequences were identified. 15 of these sequences were removed for absence of complete Gp_dh_N or Gp_dh_C domain and 13 of them harbour both domains (Additional file 1). Then, the 25 putative GAPDHs were used as queries to BLASTn against the contigs and singletons assembled from EST sequences (Additional file 2). Six of them had no matched contig or singleton, suggesting that they were not expressed in wheat. Finally, 19 GAPDHs were identified in wheat (Table 1). These *GAPDH*s genes located on chromosomes 2, 4, 6 and 7. Chromosomes 2A, 2B, 4B and 4D contained one *GAPDH*, respectively. Chromosomes 2D, 6B, 6D, 7A, 7B and 7D contained two *GAPDH*s, respectively. And chromosomes 6A contained 3 *GAPDH*s (Table 1). Meanwhile, 3 *GAPN*s that located on chromosomes 2A, 2B and 2D were identified (Table 1 and Additional file 3).

**Table 1:** GAPDH gene family in wheat

Type	Gene	Chromosome	CDS	aa	MW	pI	Contigs	Singletons
GAPDH	*TaGAPDH1*	2AL	1227	408	43.37	7.00	9	13
	*TaGAPDH2*	2BL	1206	401	42.70	7.61	8	16
	*TaGAPDH3*	6AL	1014	337	36.58	6.40	13	151
	*TaGAPDH4*	6AL	1014	337	36.59	7.05	13	151
	*TaGAPDH5*	6AS	1251	416	43.86	8.15	6	4
	*TaGAPDH6*	6BL	1077	358	39.00	8.09	13	157
	*TaGAPDH7*	6BS	1236	411	43.78	6.40	3	5
	*TaGAPDH8*	6DL	1014	337	36.61	6.67	13	155
	*TaGAPDH9*	7AL	1221	406	42.80	8.65	1	4
	*TaGAPDH10*	7AL	1062	353	38.41	7.09	11	56
	*TaGAPDH11*	7BL	1227	408	42.88	8.52	1	4
	*TaGAPDH12*	7DL	1014	337	36.53	6.67	16	82
	*TaGAPDH13*	7DL	1221	406	42.58	7.60	1	4
	*TaGAPDH14*	2DL	423	141	14.74	5.44	6	15
	*TaGAPDH15*	2DL	486	161	17.48	5.32	4	16
	*TaGAPDH16*	4BL	795	265	27.98	7.72	4	3
	*TaGAPDH17*	4DL	1068	356	38.08	9.83	1	2
	*TaGAPDH18*	6DS	894	297	31.57	6.08	2	0
	*TaGAPDH19*	7BL	621	206	22.18	6.30	7	14
GAPN	*TaGAPN1*	2DS	1491	496	53.05	6.39	3	2
	*TaGAPN2*	2BS	1491	496	53.07	6.39	3	2
	*TaGAPN3*	2AS	1317	438	46.65	6.56	3	2

The coding sequences (CDS) and protein sequences of wheat *GAPDH*s that contain both domains (*TaGAPDH1*-*13*) and *GAPN*s (*TaGAPN1*-*3*) were aligned by DNAMAN with pairwise method (Additional files 4 and 5). It showed that the 13 wheat *GAPDH* genes shared 47.04–99.7 % identity at both CDS and putative amino acid levels. For instance, *TaGAPDH1*/*2*, *TaGAPDH3*/*4*/*6*/*8*, *TaGAPDH5*/*7*, *TaGAPDH9*/*11*/*13* and *TaGAPDH10*/*12* showed high identity (≥90 %) at both CDS and protein sequence levels, respectively. Meanwhile, the 3 *GAPN*s showed low identity (<30 %) with *GAPDH*s, but high identity (>90 %) with each other (Additional file 5). Moreover, the molecular weights (MW) and isoelectric points (pI) of wheat GAPDH and GAPN proteins were calculated by ProtParam tool in ExPASy. The results revealed that the MWs of GAPDHs varied from 14.74 to 43.86 kDa, and that of GAPNs varied from 46.65 to 53.07 kDa, the pIs of GAPDHs ranged from 6.40 to 8.65 while that of GAPNs floated around 6.40 (Table 1).

**Fig. 1:**
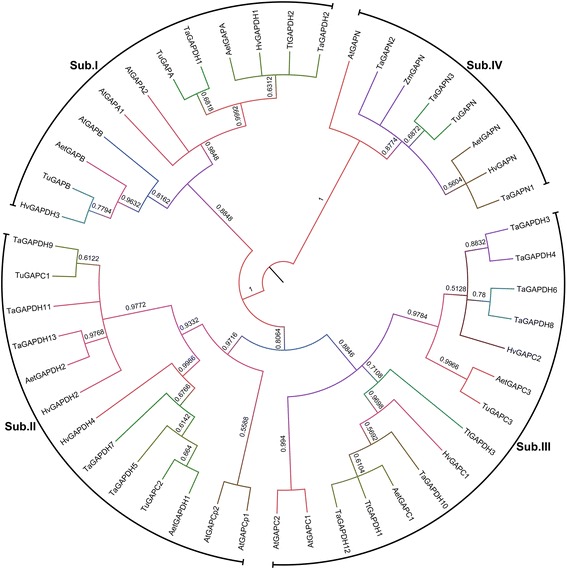
Molecular phylogenetic analysis of *GAPDH* gene families in *Arabidopsis thaliana*, *Triticum aestivum*, *Aegilops tauschii*, *Triticum urartu* and *Triticum turgidum*. The rooted Neighbor-joining phylogenetic tree of *GAPDH* genes was inferred from the amino acid sequence alignments of GAPDHs by MEGA5.1 with Neighbor-joining method under default parameters. The values on the tree represent bootstrap confidence values inferred from 1000 replicates

For phylogenetic analysis, classification and nomenclature, *Arabidopsis thaliana*, *Hordeum vulgare*, *Aegilops tauschii*, *Triticum urartu* and *Triticum turgidum GAPDH*s and GAPNs gene were identified as well (Additional file 6). And a rooted Neighbor-joining phylogenetic tree was constructed by the MEGA5.1 program with the default parameters (Fig. 1). Combing with the proposed nomenclature and subcellular location of *Arabidopsis thaliana GAPDH*s, the employed *GAPDH* genes could be distributed into four subfamilies (Fig. 1). Subfamily I was corresponding to *gapA/B* gene, subfamily II to *gapCp* genes, subfamily III to *gapC* gene, and subfamily IV to *gapN* gene. In wheat, *TaGAPDH1* and *TaGAPDH2* were distributed into subfamily I, *TaGAPDH5*/*7*/*9*/*11*/*13* were grouped into subfamily II, *TaGAPDH4*/*6*/*8*/*10*/*12* were clustered in subfamily III, and all *TaGAPN*s were in subfamily IV (Fig. 1; Additional file 6).

### Structure and conserved residues analysis of *GAPDH*

**Fig. 2:**
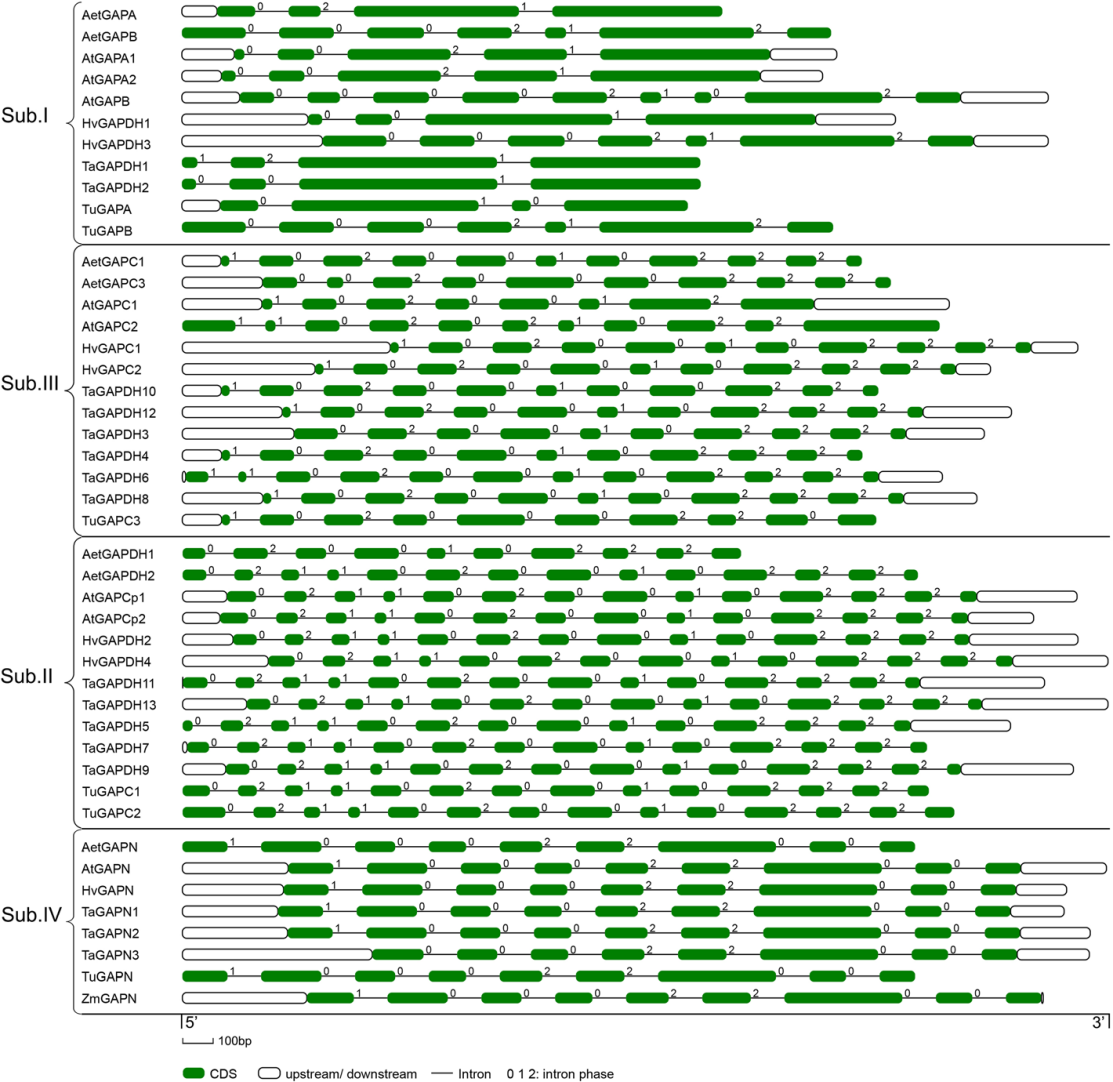
Structure analysis of *GAPDH* gene families in *Arabidopsis thaliana*, *Triticum aestivum*, *Aegilops tauschii* and *Triticum urartu*. Gene structures of *GAPDH*s analyzed by GSDS (http://gsds.cbi.pku.edu.cn/). Exons are shown as *green boxes*, introns are shown as *thin lines*, and UTRs are shown as *blank boxes*

Owing to the transcript annotation of wheat genome, the analysis and comparison of the structural features of *GAPDH*s in different families were conducted (Fig. 2). It indicated that the organization (number, order and length) of exons were almost conserved within each *GAPDH* subfamily, while the introns and UTRs showed variable lengths and distribution (Figs. 1 and 2). The majority of *GAPDH* genes in subfamilies II, III and IV contained 13, 10 and 8 introns, respectively. And in subfamily I, *GAPA*s and *GAPB*s mostly contained 8 and 4 introns, respectively. In wheat, *GAPDH*s in same subfamily shared similar exon/intron structure, for example, *TaGAPDH1*/*2*, *TaGAPDH4*/*6*/*810*/*12*, *TaGAPDH5*/*7*/*9*/*11*/*13* and *TaGAPN*s shared similar exon/intron structures, respectively (Figs. 1 and 2).

**Fig. 3:**
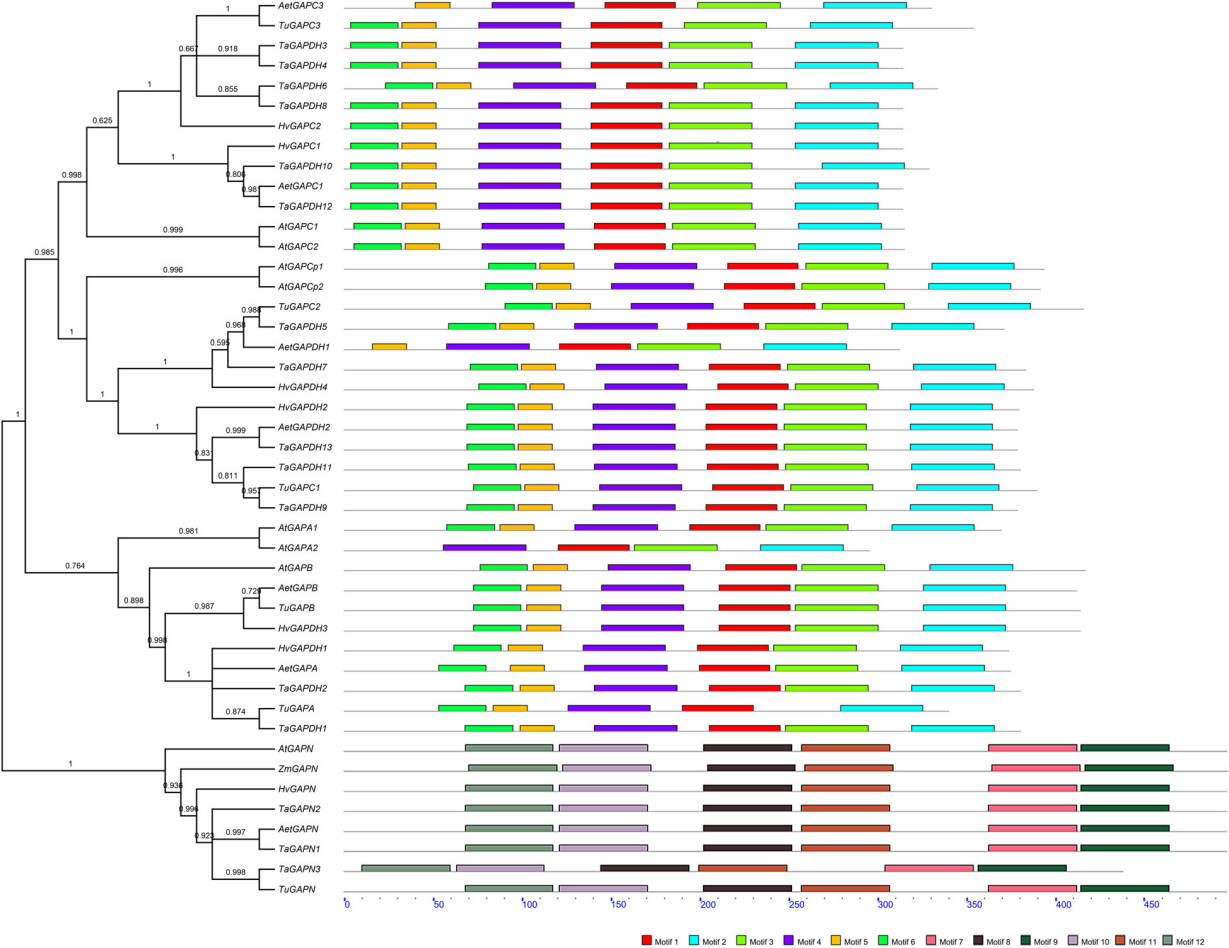
Structure of GAPDH proteins in *Arabidopsis thaliana*, *Triticum aestivum*, *Aegilops tauschii*, *Triticum urartu* and *Triticum turgidum*. The *line* represents the coding sequence and 12 motifs are shown. The amino acid sequences of GAPDH were used as an input of MEME. The total number of input *GAPDH* and *GAPN* genes is 42 and 9 respectively

To investigate the conserved residues of GAPDH proteins, we aligned wheat GAPDH protein sequences and detected the conserved sites of them (Additional file 7). The alignment displayed that amino acids structure of *TaGAPDH*s are fairly conserved and there are several identical regions such as “INGFGRIGR”, “KYD”, “GVF”, “GAKKV” “SNASCTTNCLAP”, “STGAAKAV”, “RVPT”, “VS”, “DF” and “WYDNEWGYS”. Among these regions, the “INGFGRIGR”, “SNASCTTNCLAP”, “RVPT”, “VS”, “DF”, and “WYDNEWGYS” match well with that of animal/fungi GAPDHs alignment (Additional file 8). It suggested that GAPDHs are much conserved during evolution. Furthermore, the alignment of GAPNs showed that GAPNs are nearly identical in different plants (Additional file 9). To give a visual insight to the GAPDH proteins similarity, a motif figure was constructed by submitting amino acid sequences of *GAPDH* genes to the MEME website (Fig. 3 and Additional file 10). Almost every GAPDH possessed the first six motifs while every GAPNs possessed the last six ones, the motif structure of GAPDH proteins from different species shared high similarity within each subfamily (Figs. 1 and 3). Furthermore, the conserved regions like “SNASCTTNCLAP” and “WYDNEWGYS” were exhibited completely in those motifs, meeting the results of alignment.

### Expression analysis of *GAPDH*s by microarray

**Fig. 4:**
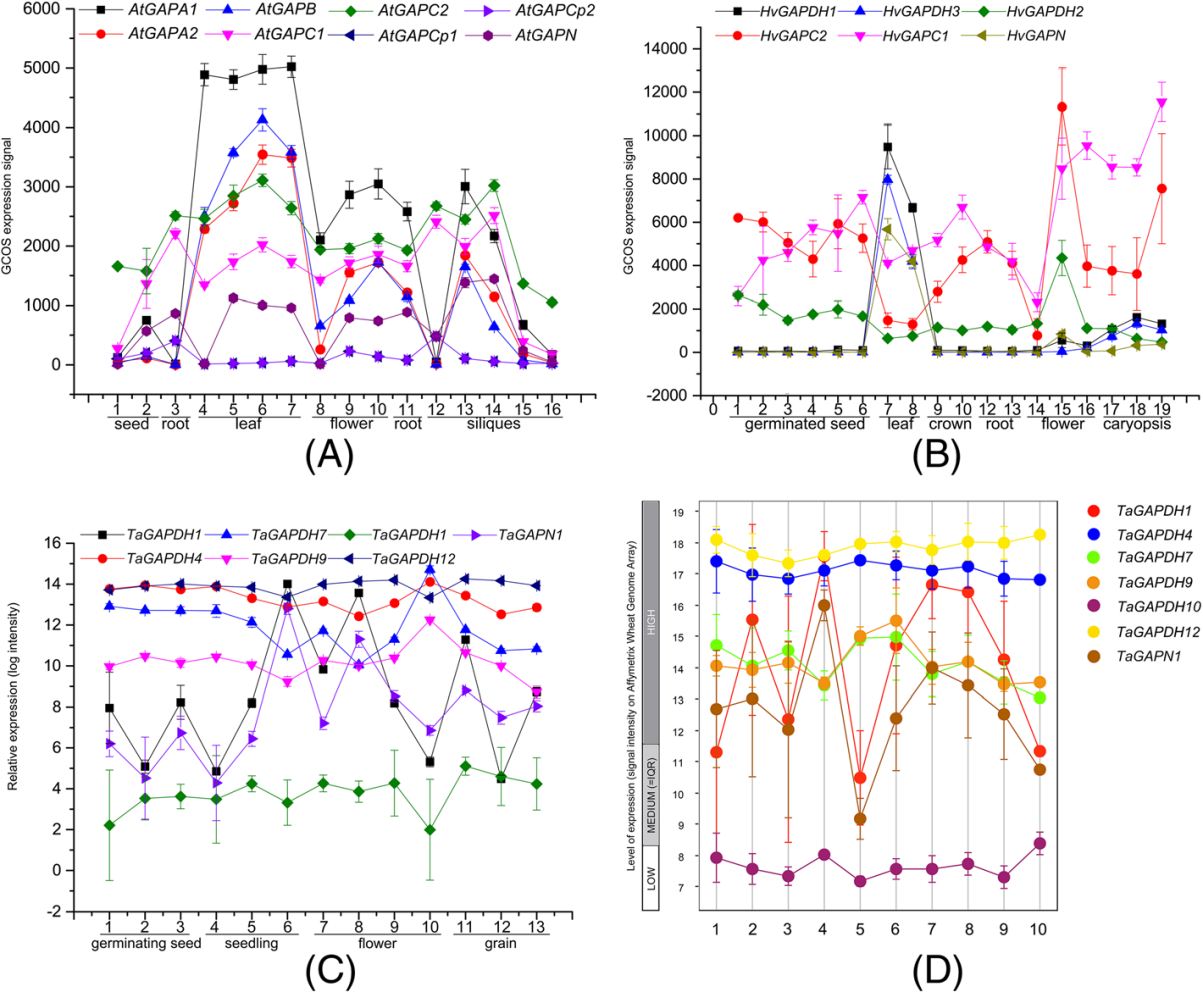
Expression profiles of *GAPDH*s in different tissues. **a** Expression profiles of *Arabidopsis thaliana GAPDH*s in different tissues. **b** Expression profiles of barley *GAPDH*s in different tissues. **c** Expression profiles of wheat *GAPDH*s in different tissues. **d** Expression profiles of wheat *GAPDH*s in different tissues at different development stages. Number 1–9 mean germination, seedling growth, tillering, stem elongation, booting, inflorescence emergence, anthesis, milk development, dough development and ripening stages, respectively

To analyze the expression profiles of *GAPDH*s, the microarray data of *Arabidopsis thaliana*, *Hordeum vulgare GAPDH*s were downloaded from the BAR website (http://bar.utoronto.ca/) with their accession numbers or corresponding probe set IDs, the expression data of wheat *GAPDH*s were downloaded from PLEXdb (http://www.plexdb.org/) (Additional file 6). Combined with the analysis on Genevestigator (https://genevestigator.com/gv/), the tissue expression profiles and the inducible expression profiles in response to stress of *GAPDH*s were generated (Fig. 4).

*GAPDH* genes were expressed almost all over the plant developmental stages and showed notable tissue specificity (Fig. 4). In Arabidopsis, *AtGAPA1*, *AtGAPA2* and *AtGAPB* were significantly highly expressed in leaf but their expression in root were negligible relative to that in leaf. *AtGAPC*s and *AtGAPN* were highly expressed in root, leaf and flower. While *AtGAPCps* showed low expression level in all tissues except relatively high level in root (Fig. 4a). In barley, *HvGAPDH1*, *HvGAPDH3* and *HvGAPN* shared the same expression pattern that significantly high in leaf but extremely low in other tissues. Meanwhile, *HvGAPDH2* slightly and nearly constantly expressed in all tissues, just as *AtGAPCp*s did (Fig. 4a and b). Then in wheat, *TaGAPDH1* and *TaGAPN1* were strongly expressed in leaf, shoot, crown and inflorescence but the case came out opposite in root. In addition, *TaGAPDH4*, *TaGAPDH7*, *TaGAPDH9*, *TaGAPDH10* and *TaGAPDH12* were expressed at roughly steady but various levels in all tissues, and among them, *TaGAPDH4*/*12* and *TaGAPDH7/9* shared synchronous expression patterns, respectively (Fig. 4c and d).

### Expression profiles of *GAPDH* genes response abiotic stresses

**Fig. 5:**
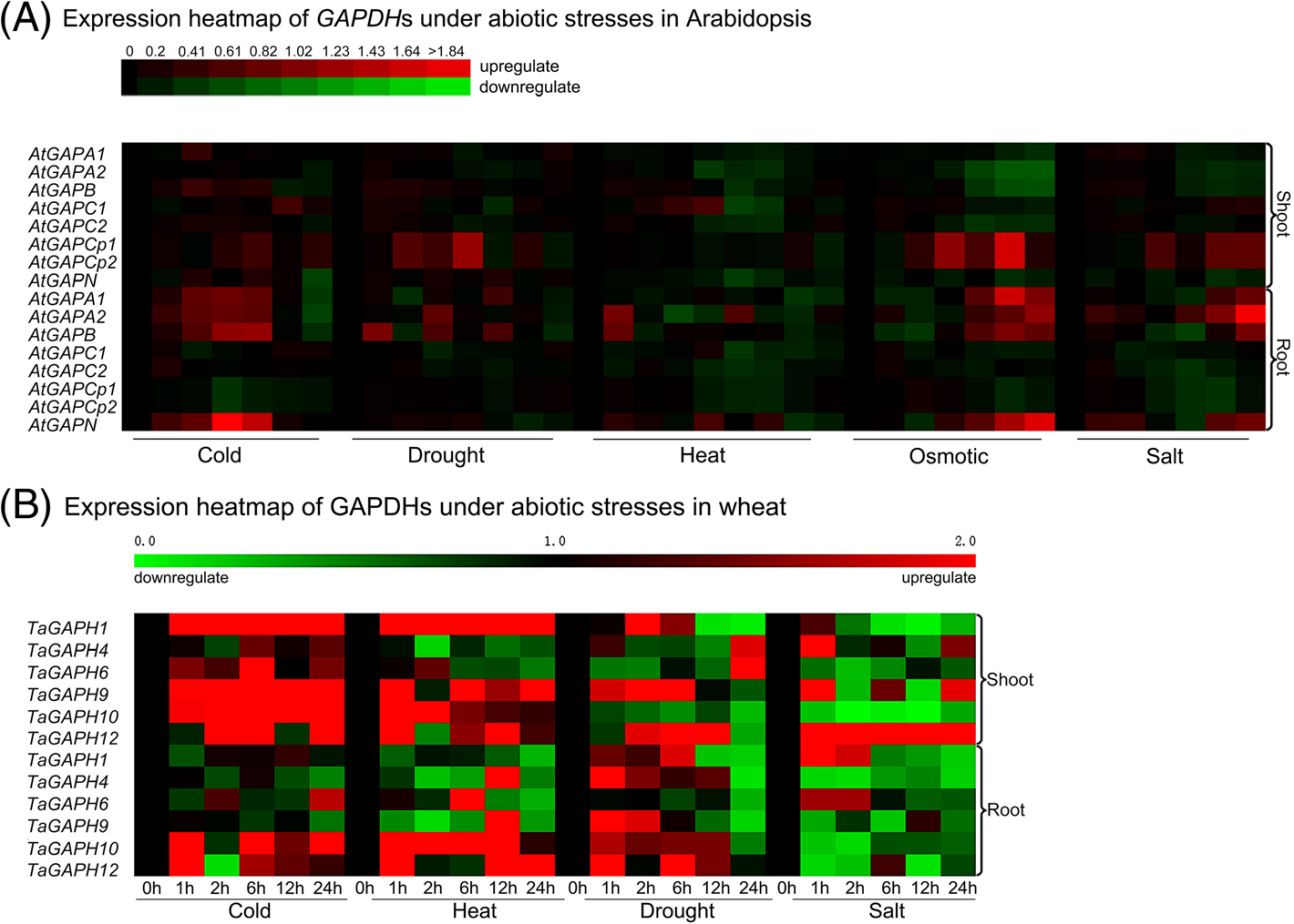
Expression analysis of *GAPDH* genes under abiotic stresses. **a** Expression heatmap of GAPDHs under abiotic stresses in Arabidopsis. The microarray data was downloaded from Arabidopsis eFP Browser. Heatmap was plot by Heatmapper. **b** Expression heatmap of GAPDHs under abiotic stresses in wheat. The data came from qRT-PCR analysis with 2^–ΔΔCt^ method. Heatmap were generated with MeV v4.9. The relative expression levels were intuitively reflected in the heatmap with the gradient color *green*/*black*/*red* (low to high)

The expression patterns of wheat *GAPDH* genes under cold, heat, drought and salt treatments were detected by qRT-PCR (Fig. 5b) and that of Arabidopsis were generated with the microarray data from Arabidopsis eFP Browser (Fig. 5a). Their expression profiles appeared to be complex in view of the results.

Under cold treatments, in Arabidopsis, *AtGAPA*s showed slightly increased expression at early stress stage in shoots but were strongly up-regulated in roots. *AtGAPB* were intensely transcribed in both shoots and roots. Roughly, *AtGAPCs* showed up-regulated expression in shoots and down-regulated expression in roots. *AtGAPCp*s shared same expression pattern: significantly up-regulated in shoots and down-regulated in roots (Fig. 5a). While in wheat, *TaGAPDH1*, *TaGAPDH6*, *TaGAPDH9*, *TaGAPDH10* and *TaGAPDH12* were dramatically up-regulated in shoots (Fig. 5b). In roots, *TaGAPDH10* and *TaGAPDH12* were up-regulated, and *TaGAPDH4* and *TaGAPDH9* were notably down-regulated (Fig. 5b).

Exposed to heat stress, transcription of most Arabidopsis *GAPDH*s were reduced in both shoots and roots except for *AtGAPB* and *AtGAPC1* were slightly up regulated in shoots (Fig. 5a). *TaGAPDH1*, *TaGAPDH9*, *TaGAPDH10* and *TaGAPDH12* were up-regulated, and *TaGAPDH4* and *TaGAPDH6* were down-regulated in shoots. In roots, most of *TaGAPDH*s were down-regulated except *TaGAPDH10* and *TaGAPDH12* (Fig. 5b).

During drought treatment, *AtGAPA*s showed down-regulated expression in shoots and fluctuant expression in roots. *AtGAPB* were up-regulated at first and down-regulated later in shoots. In roots, *AtGAPB* were intermittently up and down-regulated. *AtGAPCp*s were prominently up-regulated in shoots and transcribed without sharp variation in roots (Fig. 5a). In wheat shoots, *TaGAPDH1*, *TaGAPDH9* and *TaGAPDH12* were found up-regulated. Moreover, *TaGAPDH4*, *TaGAPDH6* and *TaGAPDH10* were down regulated. While in roots, at early stage of stress, all these *TaGAPDH*s except *TaGAPDH6* were up-regulated at the transcriptional levels (Fig. 5b).

Immersed in thick salt solution, *AtGAPCp*s in shoots and *AtGAPA*s and *AtGAPB* in roots were up regulated as the processing time increasing. Besides, the rest were down regulated (Fig. 5a). In addition, *TaGAPDH12* in wheat shoots showed high transcription levels (Fig. 5b). Furthermore, Arabidopsis *GAPDH*s expression profiles under osmotic stress were similar to that under salt treatment (Fig. 5a).

## Discussion

### Expansion of GAPDH family in wheat

Polyploidy genomes is a common phenomenon in the plant evolutionary process. Along with the genome polyploidy, the loss and insertion of large segments and the chromosomal rearrangements have occurred frequently on chromosomes, these changes might result in gene expression, silence and loss [32, 33]. Common wheat (*Triticum aestivum L*; 2n = 6x = 42; AABBDD) is allohexaploid. During its evolutionary process, two polyploidization issues had occurred. First of all, *Triticum monococcum* (2n = 2x = 14; AA) and *Aegilops speltoides* (2n = 2x = 14; SS) have hybridized and undergone a natural polyploidization and then been naturalized into *Triticum turgidum* (4n = 4x = 28; AABB). After that, *Triticum turgidum* have hybridized with *Aegilops tauschii* (2n = 2x = 14; DD) and undergone polyploidization once again resulting in the common wheat (2n = 6x = 42; AABBDD) [34, 35]. Theoretically, every wheat genes would have three homoeologous gene, but as a consequence of the side effect of polyploidy, evolutionary and acclimation process, many wheat genes have only one or two homoeologous genes that expressed. Here, 22 *GAPDH* genes representing four major types of plant *GAPDH* (*gapA*/*B*, *gapC*, *gapCp* and *gapN*) were identified in hexaploid wheat cv. *Chinese spring*, confirming the demonstration above. The high identity of wheat GAPDHs within and between subfamilies indicated that some highly identical GAPDHs may be generated via duplications (Fig. 2 and Additional file 7). Given that there were 6 and 5 *GAPDH* genes identified in *Aegilops tauschii* and *Triticum urartu* (2n = 2x = 14; AA), respectively (Table 1 and Additional file 6), we inferred that whole genome duplication and segmental duplication have contributed to expansion of *GAPDH*s family in wheat.

### GAPDHs play compelling roles in plant abiotic stress tolerance

Inducible expression analysis here revealed that those investigated *GAPDH* gene expression could be induced by at least one abiotic stress treatment (cold, heat, drought, osmotic or salt). For example, *TaGAPDH12* was found up-regulated significantly in shoots under cold, heat, drought and salt stresses (Fig. 5). It implied that GAPDH may have multiple functions except for metabolism roles in plant, such as involving in abiotic stress resistance. This novel function of GAPDH was ubiquitous in plants. For instance, maize *GAPC3* and *GAPC4* were induced by anaerobic stress [36], and overexpression of a rice cytosolic gene *OsGAPC3* enhances salt tolerance [25]. In Arabidopsis thaliana, *GAPC*s, transduced the H_2_O_2_ signal by interacting with the plasma membrane-associated phospholipase D (PLDδ) and the knockout of *GAPC*s made plants less responsive to water deficits than the wild type [27].

Taken together, it was demonstrated that GAPDH is a multifunction protein besides its key role in glycolysis, especially in plant abiotic stress resistance. Further study to detect its moonlight function and illuminate concrete mechanism will enhance the understanding of this common but amazing protein.

## Conclusions

Wheat carried 22 *GAPDH* genes, representing four types of plant *GAPDH*s (*gapA*/*B*, *gapC*, *gapCp* and *gapN*). Whole genome duplication and segmental duplication might account for the expansion of wheat *GAPDH*s. The 22 *GAPDH*s were distributed on chromosomes 2, 4, 6 and 7. According to phylogenetic analysis and structural characteristics, *GAPDH*s could be classified into four subfamilies. Microarray analysis showed that *GAPDH* genes were expressed almost all over the plant developmental stages with notable tissue specificity and involved in several abiotic stresses responses. A further qRT-PCR analysis of wheat *GAPDH*s indicated that these *GAPDH* genes followed different expression patterns in response to abiotic stresses. It was speculated that GAPDHs play roles in plants abiotic stress tolerance.

## Methods

### Database mining and identification of GAPDH genes

Wheat, barley, *Aegilops tauschii*, and *Triticum urartu* genome sequences and protein sequences were downloaded from Ensembl Plants (http://plants.ensembl.org/index.html) [34, 37–39]. A local protein database were established with these protein sequences. To investigate the *GAPDH* genes in above species, HMMER v3.0 was employed to perform an HMM search against the established local protein database [40], using the family-specific Gp_dh_N domain (PF00044) and Gp_dh_C domain (PF02800) HMM profiles obtained from the Pfam database (http://pfam.xfam.org/search) [41] as query, with the default parameters and an E-value cutoff of 1e^−5^. To refine the search results, partial GAPDH domains and other potential false positives were eliminated manually with the Pfam and CDD (http://www.ncbi.nlm.nih.gov/Structure/cdd/wrpsb.cgi) databases [42]. The information and sequences of Arabidopsis *GAPDH*s (*AtGAPDH*s) were retrieved from The Arabidopsis Information Resource (TAIR; http://www.arabidopsis.org/).

The previously reported Arabidopsis *GAPDH* cDNA sequences were used as queries to BLASTn the GenBank EST database for *Triticum aestivum* (taxid:4565) and *Triticum turgidum* (taxid:4571). The EST sequences were assembled into contigs using CodonCode Aligner with high stringency parameters of minimum percent identity of 99 %, minimum overlap length of 50 and default parameters for the rest. Open reading frames (ORF) of obtained contigs were carried out with the ORF Finder in NCBI (http://www.ncbi.nlm.nih.gov/gorf/gorf.html), then partial GAPDH domains and other potential false positives were eliminated. Basic data about the GAPDH proteins, such as amino acid number (aa), molecular weight (MW) and isoelectric point (pI), were calculated with ProtParam tool in ExPASy (http://web.expasy.org/protparam/).

### Sequence alignment and phylogenetic analysis

Multiple sequence alignment for *GAPDH*s coding sequences in Arabidopsis, *Triticum turgidum*, wheat, barley, *Aegilops tauschii*, and *Triticum urartu* were performed and edited using MEGA5.1 with the Clustal Omega method [43]. A rooted Neighbor-joining phylogenetic tree of these *GAPDH*s were constructed with MEGA5.1 under default parameters and bootstrap 1000 [43]. The coding sequences (CDS) and protein sequences of wheat *GAPDH*s were aligned by DNAMAN 6.0 software with pairwise method [44].

### Gene and protein structure analysis

To investigate the exon/intron structures of individual GAPDH gene, we aligned the coding or cDNA sequences with their corresponding genomic DNA sequences. The structure models were collected through the Gene Structure Display Server (http://gsds.cbi.pku.edu.cn) [45]. The GAPDH amino acid sequences were used as input to the MEME website (http://meme-suite.org/tools/meme) to analyze the conserved motifs and plot the motif logos [46].

### Expression profile analysis

To analyze the expression profiles of *GAPDH*s, the microarray data of *Arabidopsis thaliana*, *Hordeum vulgare GAPDH*s were downloaded from the BAR and other Data Analysis Tools for Plant Biology (http://bar.utoronto.ca/) with their accession numbers or corresponding probe set IDs and the expression data of wheat *GAPDH*s were downloaded from PLEXdb (http://www.plexdb.org/) [47]. Combined with the analysis on Genevestigator (https://genevestigator.com/gv/) [48], the tissue expression profiles and the inducible expression profiles in response to stress of *GAPDH*s were generated by software OriginPro 9.1 and Heatmapper tool in the BAR (http://bar.utoronto.ca/ntools/cgi-bin/ntools_heatmapper.cgi).

### Plant materials

Wheat seeds were sterilized with 75 % alcohol and 15 % sodium hypochlorite, rinsed 10 times, and placed on moistened filter paper in Petri dishes and germinated for 1 day and cultivated in a growth room at 22 °C and a 16 h light and 8 h dark photoperiod. Following 10 days of growth, drought and salinity, cold and heat treatments were initiated. The seedlings were grown under 4 or 40 °C for 24 h to simulate cold and heat treatments, respectively; and immersed in 250 mM NaCl or 20 % PEG8000 solutions for 24 h as salt and drought treatments, respectively. Control and treated seeding were harvested for assays at 0, 1, 2, 6, 12 h and 24 h after treatment. All samples were immediately frozen in liquid nitrogen and kept at −80 °C prior to RNA isolation. All experiments were repeated 3 times.

### Quantitative real-time PCR (qRT-PCR)

Frozen tissues were ground in liquid nitrogen and total RNA were isolated using the RNAiso plus reagent (TaKaRa, Japan) as per the manufacturer’s specifications and treated with RNase-free DNase I (Invitrogen, USA) for 15 min to degrade any residual genomic DNA. For real-time PCR analysis, first-strand cDNAs were synthesized from DNaseI-treated total RNA using PrimeScript™ RT-PCR Kit (TaKaRa, Japan) according to the manufacturer’s instructions. Real-time PCR was performed in optical 96-well plates (BIOplastics, Netherlands) with CFX96 Touch Real-Time PCR Detection System (BIO-RAD, USA) using the SYBR Green method. The wheat *β-actin* gene (AB181991) was used as an internal control. The PCR thermal cycle was set up as follows: 95 °C for 1 min; 45 cycles of 94 °C for 15 s, 56 °C for 15 s and 72 °C for 20 s. The quantitative analysis was accomplished with the 2^-△△CT^ method and the relative expression of *TaGAPDH*s were clustered by MeV v4.9 using the average linkage hierarchical clustering method [49]. The gene specific primers used for quantitative real-time RT–PCR are listed in Additional file 11. Three biological replicates were set up and all experiments were repeated 3 times.

### Availability of supporting data

The data sets supporting the results of this article are included within the article and its additional files.

## Additional files

Additional file 1: Table S1. HMMsearch hits of wheat Glyceraldehyde 3-phosphate dehydrogenase. (PDF 93 kb) Additional file 2: The contigs and singletons of wheat GAPDH that assembled from ESTs. (TXT 704 kb) Additional file 3: The contigs and singletons of wheat GAPN that assembled from ESTs. (TXT 9 kb) Additional file 4: The GAPDH and GAPN protein sequences of wheat and other species. (TXT 19 kb) Additional file 5: Table S2. Pairwise alignments of wheat Glyceraldehyde 3-phosphate dehydrogenase. (PDF 33 kb) Additional file 6: Table S3. GAPDH family in plants. (PDF 49 kb) Additional file 7: Figure S1. Multiple alignment of wheat GAPDH amino acid sequences. (PDF 462 kb) Additional file 8: Figure S2. Multiple alignment of animal/fungi/plant GAPDH amino acid sequences. (PDF 485 kb) Additional file 9: Figure S3. Multiple alignment of GPAN amino acid sequences. (PDF 345 kb) Additional file 10: Figure S4. Motif LOGOs of GAPDHs and GAPNs generated by MEME. (PDF 593 kb) Additional file 11: Table S4. Primers used in this study. (PDF 50 kb)

## Abbreviations

GAPDH: glyceraldehyde-3-phosphate dehydrogenase
G3P: glyceraldehyde-3-phosphate
GAPA/B: chloroplastic GAPDHs
GAPC: phosphorylating GAPDH in cytoplasm
GAPCp: GAPDHs in non-green plastids
GAPN: cytosolic non-phosphorylating GAPDH
NtOSAK: tobacco osmotic stress-activated protein kinase
PLDδ: phospholipase Dδ
ABA: abscisic acid
PEG: polyethylene glycerol
