# Supplementary material for: Genome-wide identification and characterization of Glyceraldehyde-3-phosphate dehydrogenase genes family in wheat (Triticum aestivum)
Source: BMC Genomics. 2016 Mar 16;17:240. doi: 10.1186/s12864-016-2527-3 (PMC4793594; doi:10.1186/s12864-016-2527-3)
Supplement: Additional file 1: Table S1. — HMMsearch hits of wheat Glyceraldehyde 3-phosphate dehydrogenase. (PDF 93 kb) [file 12864_2016_2527_MOESM1_ESM.pdf]

Table S1 HMMsearch hits of wheat Glyceraldehyde 3-phosphate dehydrogenase

	Hits	Gp_dh_N		Gp_dh_C		Adledh	
		from	to	from	to	from	to
	Traes_1AS_24D49363B.1	42	151	1	158	–	–
	Traes_1BS_E12ED9754.1	128	151	1	52	–	–
	Traes_2AL_783CF383F.1	2	151	1	158	–	–
	Traes_2BL_5D64E8C87.1	2	151	1	158	–	–
	Traes_2DL_04AA47AA7.1	22	151	–	–	–	–
	Traes_2DL_80DD45E69.1	2	19	–	–	–	–
	Traes_2DS_8C0F16AC1.1	76	109	–	–	–	–
	Traes_2DL_93867FC79.1	–	–	19	158	–	–
	Traes_4BL_A9FAA75A9.1	2	151	–	–	–	–
	Traes_4BL_F32809B15.1	–	–	109	158	–	–
	Traes_4BS_BCAC4E57B.18	–	–	1	61	–	–
	Traes_4DL_06EF3E3A2.1	33	151	1	133	–	–
	Traes_4DL_5612CF456.2	2	151	1	28	–	–
	Traes_4DS_19608B1FF.1	2	123	–	–	–	–
	Traes_4DL_F8980B1D2.1	–	–	132	158	–	–
	Traes_4DS_0426CF8E9.1	–	–	123	158	–	–
	Traes_4DS_2F98108D7.1	93	151	–	–	–	–
	Traes_4DS_5E720DFF7.1	40	151	1	158	–	–
GAPDH	Traes_4DS_A3B293612.1	40	151	1	91	–	–
	Traes_4DS_D9999817B.1	2	146	–	–	–	–
	Traes_4DS_DD602A86C.1	–	–	106	158	–	–
	Traes_4DS_E5AD7818A.1	–	–	19	158	–	–
	Traes_4DS_EC8115DE9.1	–	–	90	158	–	–
	Traes_5AL_C309589A2.1	30	68	–	–	–	–
	Traes_5BS_3E6223216.1	2	93	–	–	–	–
	Traes_6AL_174253A75.2	6	151	1	158	–	–
	Traes_6AL_174253A751.1	1	151	1	158	–	–
	Traes_6AS_D1274A812.1	2	151	1	158	–	–
	Traes_6BL_B95E66E93.1	1	151	1	158	–	–
	Traes_6BS_970784007.1	2	151	1	158	–	–
	Traes_6DL_B3AD4834A.1	1	151	1	158	–	–
	Traes_6DS_5F1004AAF.2	39	151	1	158	–	–
	Traes_7AL_226028C17.1	2	151	1	158	–	–
	Traes_7AL_D93FC054C.1	1	151	1	158	–	–
	Traes_7BL_409D8E4DD.1	76	151	2	62	–	–
	Traes_7BL_A38105EC0.1	2	151	1	158	–	–
	Traes_7BL_D5E233F60.1	128	151	1	158	–	–

	Traes_7BL_E09E4240E.1	128	151	1	59	–	–
	Traes_7DL_961822B36.2	1	151	1	158	–	–
	Traes_7DL_ADFCB28B3.1	2	151	1	158	–	–
GAPN	Traes_2DS_22500FA4F.1	–	–	–	–	1	496
	Traes_2BS_8684E0E15.1	–	–	–	–	1	496
	Traes_2AS_A4A5BE528.1	–	–	–	–	59	496

The hits that are absence of complete Gp_dh_N and Gp_dh_C were colored in yellow and excluded.
