# Supplementary material for: Genome-wide identification and characterization of Glyceraldehyde-3-phosphate dehydrogenase genes family in wheat (Triticum aestivum)
Source: BMC Genomics. 2016 Mar 16;17:240. doi: 10.1186/s12864-016-2527-3 (PMC4793594; doi:10.1186/s12864-016-2527-3)
Supplement: Additional file 5: Table S2. — Pairwise alignments of wheat Glyceraldehyde 3-phosphate dehydrogenase. (PDF 33 kb) [file 12864_2016_2527_MOESM5_ESM.pdf]

Table S2 Pairwise alignments of wheat Glyceraldehyde 3-phosphate dehydrogenase

Nucleotide identity / Protein	*TaGapdh1*	*TaGapdh2*	*TaGapdh3*	*TaGapdh4*	*TaGapdh5*	*TaGapdh6*	*TaGapdh7*	*TaGapdh8*	*TaGapdh9*	*TaGapdh10*	*TaGapdh11*	*TaGapdh12*	*TaGapdh13*	*TaGapn1*	*TaGapn2*	*TaGapn3*
TaGAPDH1		98.67	53.94	54.84	47.04	53.66	49.37	55.23	47.12	57.21	47.16	57.11	48.57	35.34	36.53	35.15
TaGAPDH2	98.28		53.94	55.03	49.20	54.22	49.03	55.53	48.16	57.51	47.1	57.41	48.70	35.85	37.91	37.07
TaGAPDH3	36.99	36.99		98.32	65.58	96.94	65.48	96.94	66.37	80.28	66.96	80.87	66.57	38.88	37.58	37.20
TaGAPDH4	63.33	64.43	97.99		66.47	98.52	66.37	98.62	67.46	81.36	67.85	81.95	67.66	38.88	38.66	37.02
TaGAPDH5	72.90	72.32	65.45	70.74		64.43	92.31	66.07	82.39	67.46	83.29	67.56	80.38	36.82	35.67	34.94
TaGAPDH6	65.28	67.91	97.20	93.85	74.1		64.24	99.31	65.51	81.85	65.86	82.54	65.89	36.48	39.37	37.08
TaGAPDH7	72.90	73.19	60.98	71.36	94.71	74.76		66.27	79.67	67.06	80.21	67.46	79.69	33.88	35.23	34.42
TaGAPDH8	63.08	64.18	97.59	99.7	70.02	94.13	70.63		67.36	81.56	67.75	82.25	67.56	37.07	38.06	36.73
TaGAPDH9	71.95	73.17	61.38	72.97	90.63	76.41	91.28	72.73		66.27	96.96	66.57	96.81	35.29	35.54	34.41
TaGAPDH10	61.08	62.11	76.42	91.22	69.28	86.36	69.86	91.50	69.98		66.86	97.63	66.57	35.00	36.67	35.15
TaGAPDH11	72.02	73.97	61.13	72.86	91.83	75.79	91.55	72.62	97.79	70.59		66.96	96.72	35.86	36.19	36.43
TaGAPDH12	64.06	65.17	76.83	94.66	72.18	89.39	72.82	94.96	72.97	95.18	73.59		66.67	35.19	35.81	33.57
TaGAPDH13	72.44	73.17	60.57	73.22	91.11	76.41	91.77	72.97	98.52	70.45	96.81	73.46		35.13	34.38	33.99
TaGAPN1	18.15	18.15	15.73	15.79	13.25	16.20	15.94	16.86	18.09	18.37	18.82	16.04	21.08		98.26	97.65
TaGAPN2	13.24	13.24	16.67	16.67	18.08	16.67	13.53	16.67	18.97	19.90	18.28	17.04	17.53	99.42		97.42
TaGAPN3	20.83	20.83	16.23	16.23	17.55	16.23	15.64	16.23	17.41	12.60	18.94	18.41	14.79	97.31	99.32	

The number above the dash line are the identity between CDS sequences, and the number below the dash line represent the identity between amino acid sequences.
