# Supplementary material for: Genome-wide identification and characterization of Glyceraldehyde-3-phosphate dehydrogenase genes family in wheat (Triticum aestivum)
Source: BMC Genomics. 2016 Mar 16;17:240. doi: 10.1186/s12864-016-2527-3 (PMC4793594; doi:10.1186/s12864-016-2527-3)
Supplement: Additional file 6: Table S3. — GAPDH family in plants. (PDF 49 kb) [file 12864_2016_2527_MOESM6_ESM.pdf]

Table S3 GAPDH family in plants

Organism	Gene ID	Nomenclature	Subfamily
Triticum aestivum	Traes_2AL_783CF383F	*TaGAPDH1*	Sub.I
	Traes_2BL_5D64E8C87	*TaGAPDH2*	Sub.I
	Traes_6AL_174253A75	*TaGAPDH3*	Sub.III
	Traes_6AL_174253A751	*TaGAPDH4*	Sub.III
	Traes_6AS_D1274A812	*TaGAPDH5*	Sub.II
	Traes_6BL_B95E66E93	*TaGAPDH6*	Sub.III
	Traes_6BS_970784007	*TaGAPDH7*	Sub.II
	Traes_6DL_B3AD4834A	*TaGAPDH8*	Sub.III
	Traes_7AL_226028C17	*TaGAPDH9*	Sub.II
	Traes_7AL_D93FC054C	*TaGAPDH10*	Sub.III
	Traes_7BL_A38105EC0	*TaGAPDH11*	Sub.II
	Traes_7DL_961822B36	*TaGAPDH12*	Sub.III
	Traes_7DL_ADFCB28B3	*TaGAPDH13*	Sub.II
	Traes_2DS_22500FA4F	*TaGAPN1*	Sub.IV
	Traes_2BS_8684E0E15	*TaGAPN2*	Sub.IV
	Traes_2AS_A4A5BE528	*TaGAPN3*	Sub.IV
Arabidopsis thaliana	AT3G26650	*AtGAPA1*	Sub.I
	AT1G12900	*AtGAPA2*	Sub.I
	AT1G42970	*AtGAPB*	Sub.I
	AT3G04120	*AtGAPC1*	Sub.III
	AT1G13440	*AtGAPC2*	Sub.III
	AT1G79530	*AtGAPCp1*	Sub.II
	AT1G16300	*AtGAPCp2*	Sub.II
	AT2G24270	*AtGAPN*	Sub.IV
Aegilops tauschii	EMT31124	*AetGAPA*	Sub.I
	EMT25216	*AetGAPC3*	Sub.III
	EMT07643	*AetGAPDH1*	Sub.II
	EMT03645	*AetGAPDH2*	Sub.II
	EMT23312	*AetGAPC1*	Sub.III
	EMT30317	*AetGAPB*	Sub.I
	EMT24014	*AetGAPN*	Sub.IV
Hordeum vulgare	MLOC_44511.1	*HvGAPDH1*	Sub.I
	MLOC_72170.1	*HvGAPC2*	Sub.III
	MLOC_52515.2	*HvGAPDH3*	Sub.I
	MLOC_18233.1	*HvGAPC1*	Sub.III
	MLOC_66206.1	*HvGAPDH2*	Sub.II
	MLOC_15296.1	*HvGAPDH4*	Sub.II
	MLOC_77485.3	*HvGAPN*	Sub.IV
Triticum urartu	TRIUR3_15760-T1	*TuGAPA*	Sub.I
	TRIUR3_34401-T1	*TuGAPC3*	Sub.III

	TRIUR3_33766-T1	*TuGAPC2*	Sub.II
	TRIUR3_06352-T1	*TuGAPC1*	Sub.II
	TRIUR3_19023-T1	*TuGAPB*	Sub.I
Triticum turgidum	N/A	*TtGAPDH1*	Sub.III
	N/A	*TtGAPDH2*	Sub.I
	N/A	*TtGAPDH3*	Sub.III

GAPDHs in *Triticum turgidum* were identified by EST sequence assembly thus thei available. Probesets of *Aegilops tauschii*, *Triticum urartu* and *Triticum turgidum* G available in PLEXdb.

**Probeset**
Ta.30808.1.S1_s_at
Ta.30808.1.S1_s_at
Ta.24991.1.S1_x_at
Ta.24991.1.S1_x_at
Ta.10213.1.S1_at
Ta.24991.1.S1_x_at
Ta.10213.1.S1_at
Ta.24991.1.S1_x_at
Ta.9557.1.A1_at
Ta.28672.1.S1_at
Ta.9557.1.A1_at
Ta.24977.1.S1_x_at
Ta.9557.1.A1_at
Ta.511.1.S1_at
Ta.511.1.S2_at
Ta.511.1.S3_at
—
—
—
—
—
—
—
—
N/A
N/A
N/A
N/A
N/A
N/A
N/A
—
—
—
—
—
—
—
N/A
N/A

N/A
N/A
N/A
N/A
N/A
N/A

ir Gene IDs are not
APDHs are not
