# Supplementary material for: Genome-wide identification and characterization of Glyceraldehyde-3-phosphate dehydrogenase genes family in wheat (Triticum aestivum)
Source: BMC Genomics. 2016 Mar 16;17:240. doi: 10.1186/s12864-016-2527-3 (PMC4793594; doi:10.1186/s12864-016-2527-3)
Supplement: Additional file 7: Figure S1. — Multiple alignment of wheat GAPDH amino acid sequences. (PDF 462 kb) [file 12864_2016_2527_MOESM7_ESM.pdf]

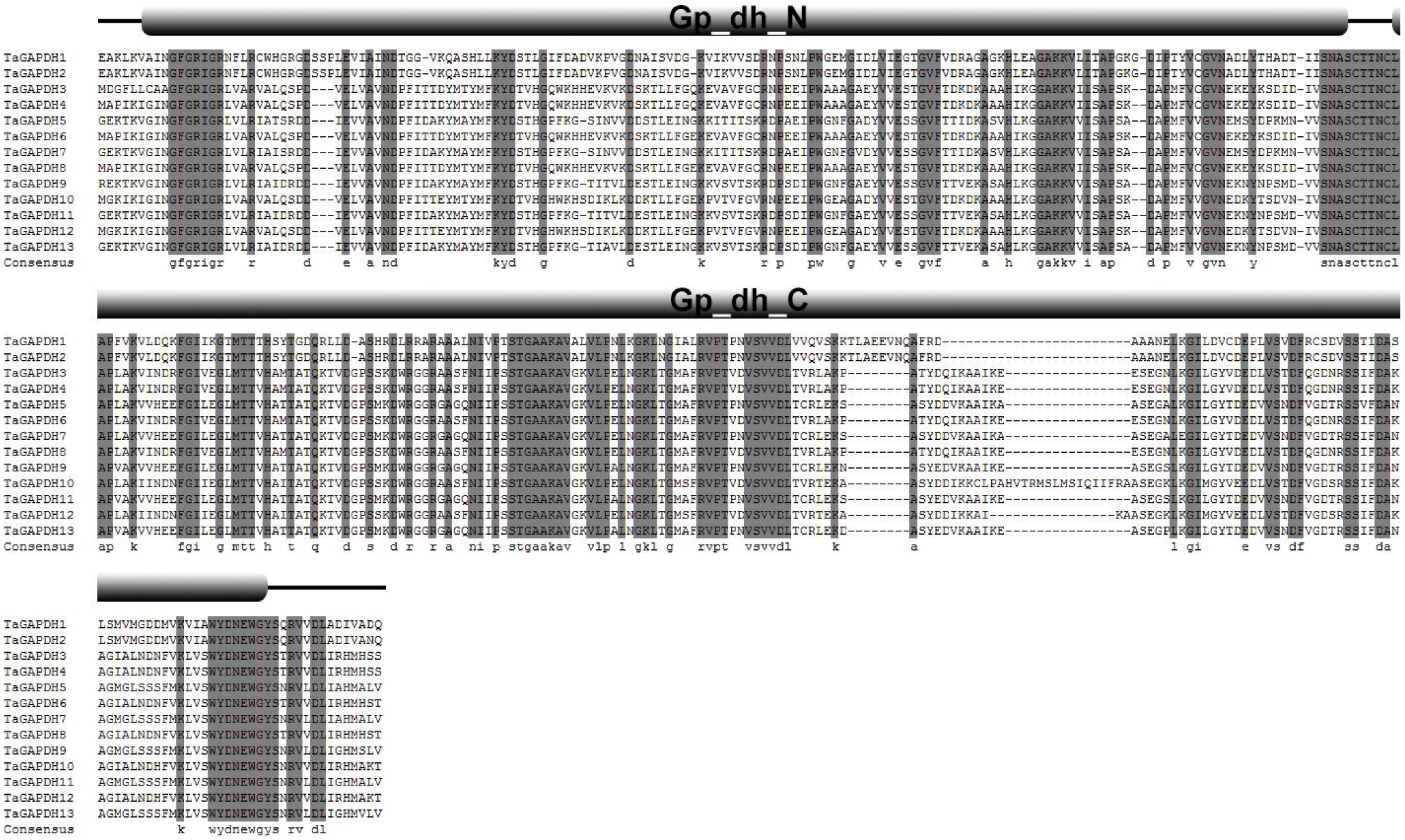

Figure S1 **Multiple alignment of wheat GAPDH amino acid sequences.** The alignment were trimmed manually and the results were generated by DNAMAN. The identical sequences are displayed in gray.
