# Supplementary material for: Genome-wide identification and characterization of Glyceraldehyde-3-phosphate dehydrogenase genes family in wheat (Triticum aestivum)
Source: BMC Genomics. 2016 Mar 16;17:240. doi: 10.1186/s12864-016-2527-3 (PMC4793594; doi:10.1186/s12864-016-2527-3)
Supplement: Additional file 8: Figure S2. — Multiple alignment of animal/fungi/plant GAPDH amino acid sequences. (PDF 485 kb) [file 12864_2016_2527_MOESM8_ESM.pdf]

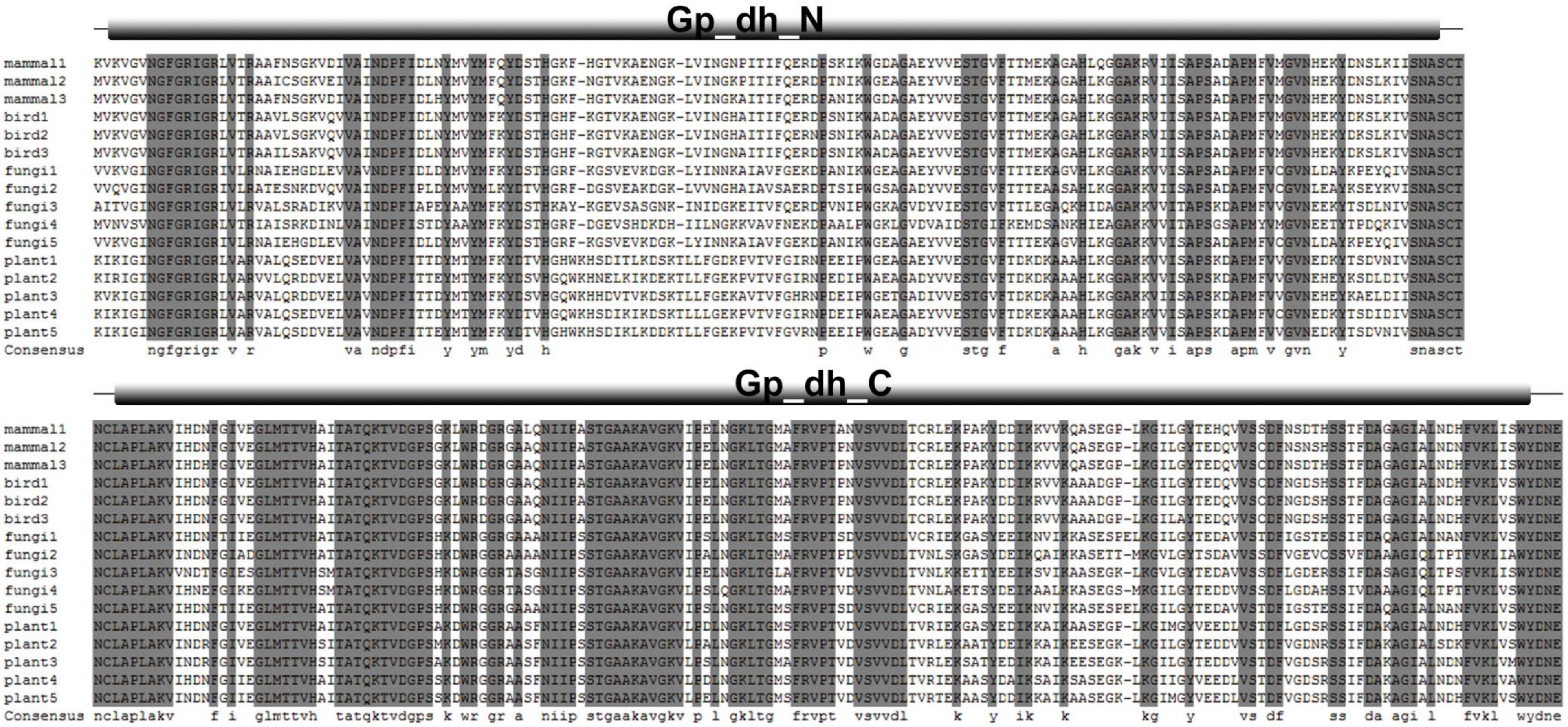


**Figure S2 Multiple alignment of animal/fungi/plant GAPDH amino acid sequences.** The alignment were trimmed manually and the results were generated by DNAMAN. The identical sequences are displayed in gray. The accessions of these GAPDHs are P04406.3, P16858.2, P00355.4, P00356.3, NP_001290108.1, O57479.3, P0CN74.1, CAC37403.1, Q92263.1, P08439.1, P0CN75.1, CAA33620.1, P25858.2, AGV54256.1, Q0J8A4.1, CAA42901.1, successively.
