# Supplementary material for: Genome-wide identification and characterization of Glyceraldehyde-3-phosphate dehydrogenase genes family in wheat (Triticum aestivum)
Source: BMC Genomics. 2016 Mar 16;17:240. doi: 10.1186/s12864-016-2527-3 (PMC4793594; doi:10.1186/s12864-016-2527-3)
Supplement: Additional file 10: Figure S4. — Motif LOGOs of GAPDHs and GAPNs generated by MEME. (PDF 593 kb) [file 12864_2016_2527_MOESM10_ESM.pdf]

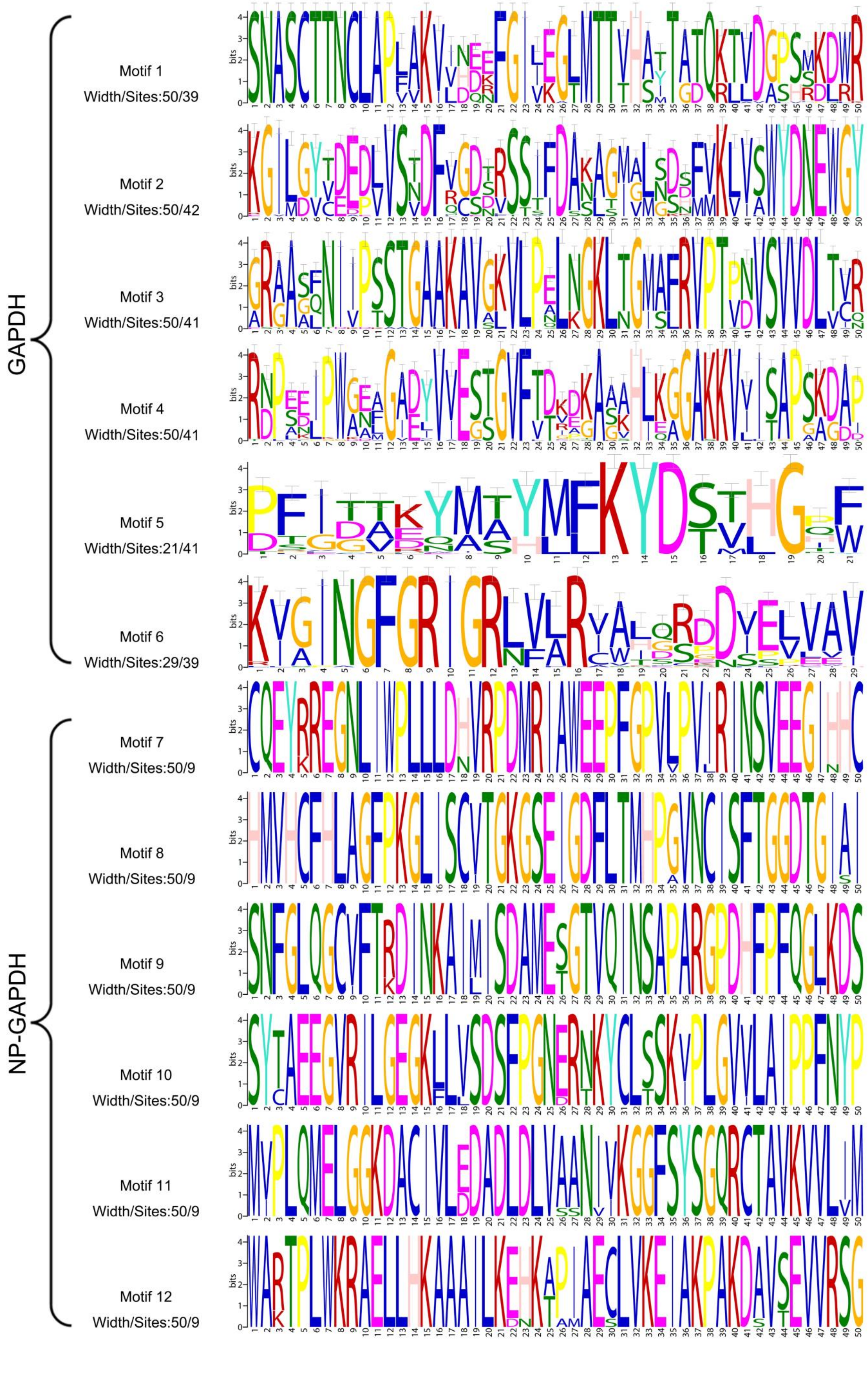
GAPDH
Motif 1
Width/Sites:50/39
Motif 2
Width/Sites:50/42
Motif 3
Width/Sites:50/41
Motif 4
Width/Sites:50/41
Motif 5
Width/Sites:21/41
Motif 6
Width/Sites:29/39
NP-GAPDH
Motif 7
Width/Sites:50/9
Motif 8
Width/Sites:50/9
Motif 9
Width/Sites:50/9
Motif 10
Width/Sites:50/9
Motif 11
Width/Sites:50/9
Motif 12
Width/Sites:50/9

Figure S4 Motif LOGOs of GAPDHs and GAPNs generated by MEME. The amino acid sequences of GAPDH were used as an input of MEME and the sites means counts of *GAPDH* genes harbors this motif and the width represents the length of motif. The maximum motif width was set as 50 here.
