# Supplementary material for: Genome-wide identification and characterization of Glyceraldehyde-3-phosphate dehydrogenase genes family in wheat (Triticum aestivum)
Source: BMC Genomics. 2016 Mar 16;17:240. doi: 10.1186/s12864-016-2527-3 (PMC4793594; doi:10.1186/s12864-016-2527-3)
Supplement: Additional file 11: Table S4. — Primers used in this study. (PDF 50 kb) [file 12864_2016_2527_MOESM11_ESM.pdf]

Table S4 Primers used in this study.

**Gene Name**	**Primer Sequences**	**Amplicon Length**
TaGAPDH1	F:5'-GACACCATCATCAGCAAC-3'<br>R:5'-ATGATGCCGAACTTTTGG-3'	83
TaGAPDH4	F:5'-TGCGGCTTCCTCTTCATC-3'<br>R:5'-TTCGTCTTCTCTCCACTCAAA-3'	102
TaGAPDH6	F:5'-CCTTCATCACCACCGACT-3'<br>R:5'-TACTCAGCACCAGCAGCA-3'	181
TaGAPDH9	F:5'-CCACCAGCCGTCCCACAATA-3'<br>R:5'-GAACCAATCTCCCAATCCGTC-3'	82
TaGAPDH10	F:5'-GTATGTCTTTCCGGGTTCC-3'<br>R:5'-AGGCAGGCACTTCTTGAT-3'	101
TaGAPDH12	F:5'-TGTGGATGTGTCAGTTGT-3'<br>R:5'-CAGCCTTGATAGCCTTCT-3'	83
β-actin	F:5'-CGACTCTGGTGATGGTGTGAG-3'<br>R:5'-AGCAAGGTCCAAACGAAGGA-3'	85
